# Should we treat patients with only one set of positive blood cultures for extensively drug-resistant *Acinetobacter baumannii* the same as multiple sets?

**DOI:** 10.1371/journal.pone.0180967

**Published:** 2017-07-07

**Authors:** Aristine Cheng, Yu-Chung Chuang, Hsin-Yun Sun, Chia-Jui Yang, Hou-Tai Chang, Jia-Ling Yang, Wang-Huei Sheng, Yee-Chun Chen, Shan-Chwen Chang

**Affiliations:** 1 Department of Internal Medicine, National Taiwan University Hospital Main Branch, and National Taiwan University College of Medicine, Taipei, Taiwan; 2 Graduate Institute of Clinical Medicine, College of Medicine, National Taiwan University, Taipei, Taiwan; 3 Department of Internal Medicine, Far Eastern Memorial Hospital, New Taipei City, Taiwan; 4 Department of Internal Medicine, National Taiwan University Hospital Yunlin Branch, Yunlin, Taiwan; University of Hong Kong, HONG KONG

## Abstract

*Acinetobacter* species are not considered skin commensals and under-treatment is an overriding concern when caring for critically-ill patients who are mostly at risk of extensively drug-resistant *Acinetobacter baumannii* (XDRAB) infections. Hence even a single blood culture yielding XDRAB will tend to prompt intervention. However, field observations suggest that patients with single-positive blood cultures had milder disease and were more likely to be recruited in interventional studies than those with multiple-positive blood cultures, yet no distinction is made in current clinical or trial recruitment practices. To our knowledge, this is the first study to compare the clinical characteristics and outcomes of patients with single-positive versus multiple-positive blood cultures for XDRAB. In this multicenter prospective cohort study of XDRAB bacteremic patients from July 2010 to June 2015, only patients with at least two simultaneously drawn blood cultures were included. The patients were classified as having single-positive or multiple-positive blood cultures according to the number of positive blood cultures yielding XDRAB. The primary end-point was the 28-day mortality. Of a total of 155 patients enrolled, 69 had a single-positive and 86 had multiple-positive blood cultures. Leukopenia (37.2% vs. 16.2%; *P* = 0.004), thrombocytopenia (56.0% vs. 26.5%; *P* < 0.001), higher Pitt bacteremia scores (6.6 vs. 5.5, *P* = 0.03) and higher 28-day mortality rates (70.9% vs. 43.5%; *P* = 0.001) distinguished patients with multiple-positive from those with single-positive cultures. Multivariate logistic regression showed that multi-positivity independently predicted 28-day mortality (adjusted odds ratio, 2.34; 95% confidence interval (CI), 1.03–5.28; *P* = 0.04) and the Cox regression confirmed that multi-positivity (adjusted hazard ratio, 1.80; 95% CI, 1.13–2.85; *P* = 0.01) predicted rapid mortality. Patients with multiple versus single positive blood cultures yielding XDRAB had greater morbidity and mortality. Investigators and clinicians should be aware that the blood culture positivity rate impacts outcomes of XDRAB bacteremia.

## Introduction

Extensively drug-resistant *Acinetobacter baumannii* (XDRAB) bacteremia usually occurs in severely ill patients in the intensive care unit with limited therapeutic options [1, 2]. The associated crude and attributable mortality rates are high, ranging from 41% to 69% and 10% to 43%, respectively [3–7]. Several complicated risk factors relating to initial disease severity, antimicrobial resistance, host factors, and ineffective antimicrobial therapy have been identified [7–15]. However, few studies have focused on the impact of bacterial factors. Currently identified factors such as the *Acinetobacter* genospecies and serum OXA-51 DNA load require molecular tools too sophisticated for daily clinical practice [16, 17]. In contrast, the blood culture positivity rate is an easy observation that has proven to be a semi-quantitative measure of the magnitude of *Staphylococcus aureus* bacteremia and to predict mortality in such patients [18]. However, the prognostic validity of the blood culture positivity rate is not universally applicable, as demonstrated by the similar clinical outcomes of patients with single versus multiple-positive blood cultures of enterococci or coagulase-negative staphylococci (CoNS) [19, 20].

For XDRAB bacteremia, data on the impact of the blood culture positivity rate on outcomes are lacking. Furthermore, the significance of a single positive blood culture with XDRAB is not known [21]. While physicians may clinically suspect some patients to have pseudobacteremia if only one of at least two sets of blood cultures is positive for XDRAB, the lack of diagnostic criteria that has been validated in the setting of XDRAB, and the overriding concerns of under treatment when caring for high-risk patients, makes withholding treatment difficult. In clinical practice, this may result in unnecessary therapy, further selection of resistant pathogens, longer hospitalization and increased costs. In clinical research, the lack of distinction between contamination and true bacteremia may contribute to misleading mortality rates and inferred treatment responses.

For these reasons, we compared the clinical characteristics and outcomes of patients with a single versus multiple sets of blood cultures yielding XDRAB in this prospective multicenter cohort study. We hypothesized that the mortality associated with XDRAB bacteremia is not only impacted by the patients’ characteristics, the primary infection foci, disease severity, antimicrobial susceptibilities, appropriate therapy, but also by the culture positivity rate. Our aims were to explore the clinical significance of a single-positive blood culture for XDRAB and to establish whether the total number of positive blood cultures could serve as a simple predictor of mortality for patients with XDRAB bacteremia.

## Materials and methods

### Hospital settings

This study was conducted at three hospitals in Taiwan: the National Taiwan University Hospital (NTUH), a 2200-bed medical center located in Taipei; the Far Eastern Memorial Hospital, a 1000-bed medical center in New Taipei City and the NTUH Yun-lin Branch, a 600-bed teaching hospital in Dou-liou. The Research Ethics Committees of the National Taiwan University Hospital Main Branch, Taipei, the National Taiwan University Hospital Yun-Lin Branch, Dou-Liou (201008047R) and the Far Eastern Memorial Hospital, New Taipei (100132-F) approved this study. Informed consent was waived for this observational study.

### Patients, inclusion and exclusion criteria and definitions

Adults with *A*. *baumannii* bacteremia were prospectively followed from July 2010 to June 2015. Only patients with XDRAB defined as *A*. *baumannii* non-susceptible to all drug classes except for colistin and tigecycline were included. Patients who were not admitted to hospital, patients who were less than 18 years old, or had only one set of blood cultures taken were excluded. One set of blood culture comprised an aerobic and an anaerobic bottle. Single-positive blood culture was defined as patients with multiple sets of blood culture drawn simultaneously, but only one set grew *A*. *baumannii*. Multiple-positive blood cultures were defined as 2 or more sets of blood culture yielding *A*. *baumannii* among the multiple simultaneous sets of blood culture.

Demographic data, underlying diseases, the site of infection, severity of illness, treatment, and outcomes were recorded. Prior antimicrobial use was defined by the use of the parenteral form of the antimicrobial agent in the 28 days preceding bacteremia. The sites of primary infection were identified according to definitions of the US Centers for Disease Control and Prevention (CDC) [22]. If no focus could be identified, the bacteremia was classified as primary bacteremia. The Charlson comorbidity index was used to adjust for underlying conditions [23]. Bacteremia severity was assessed using the Pitt bacteremia score at bacteremia onset [24]. Use of immunosuppressive agents was defined as the receipt of antineoplastic drugs or other immunosuppressive agents within 6 weeks or receipt of corticosteroids at a dosage ≥20 mg of prednisolone daily for ≥2 weeks or 30 mg of prednisolone daily for ≥1 week before bacteremia onset [16]. Early mortality was defined as death that occurred before or on the day of culture report. The primary outcome was the 28-day in-hospital mortality.

### Microbiological studies

Blood cultures were processed by the clinical microbiology laboratory. The *A*. *baumannii* complex was identified by the Vitek 2 identification system (bioMérieux Inc.). The antimicrobial susceptibility were determined using Vitek 2 identification system (bioMérieux Inc.) and were interpreted according to the CLSI criteria [25] After collecting the isolates, the genospecies were identified according to the 16S–23S ribosomal RNA (rRNA) gene intergenic spacer region, as described [26]. Patients with bacteremia caused by *A*. *nosocomialis* or *A*. *pittii* were excluded [16]. Only the genospecies *A*. *baumannii* was included.

### Statistical analysis

Mean and standard deviation (SD) were calculated for continuous variables and percentages for categorical variables. The Student’s *t* test and Fisher’s exact test were used to compare continuous and categorical variables, respectively. Multivariate logistic regression and Cox’s proportional-hazards models were applied for outcome analysis. All variables with a *P* value ≤0.2 in the univariate regression were considered in the multivariate analysis. Multivariable models were developed by minimizing Akaike's information criterion [27]. A sensitivity analysis excluding polymicrobial-infected patients was conducted. Analysis was performed using Stata software (version 14, StataCorp, College Station, TX). Two-sided *P* values ≤0.05 were considered significant.

## Results

**Fig 1:**
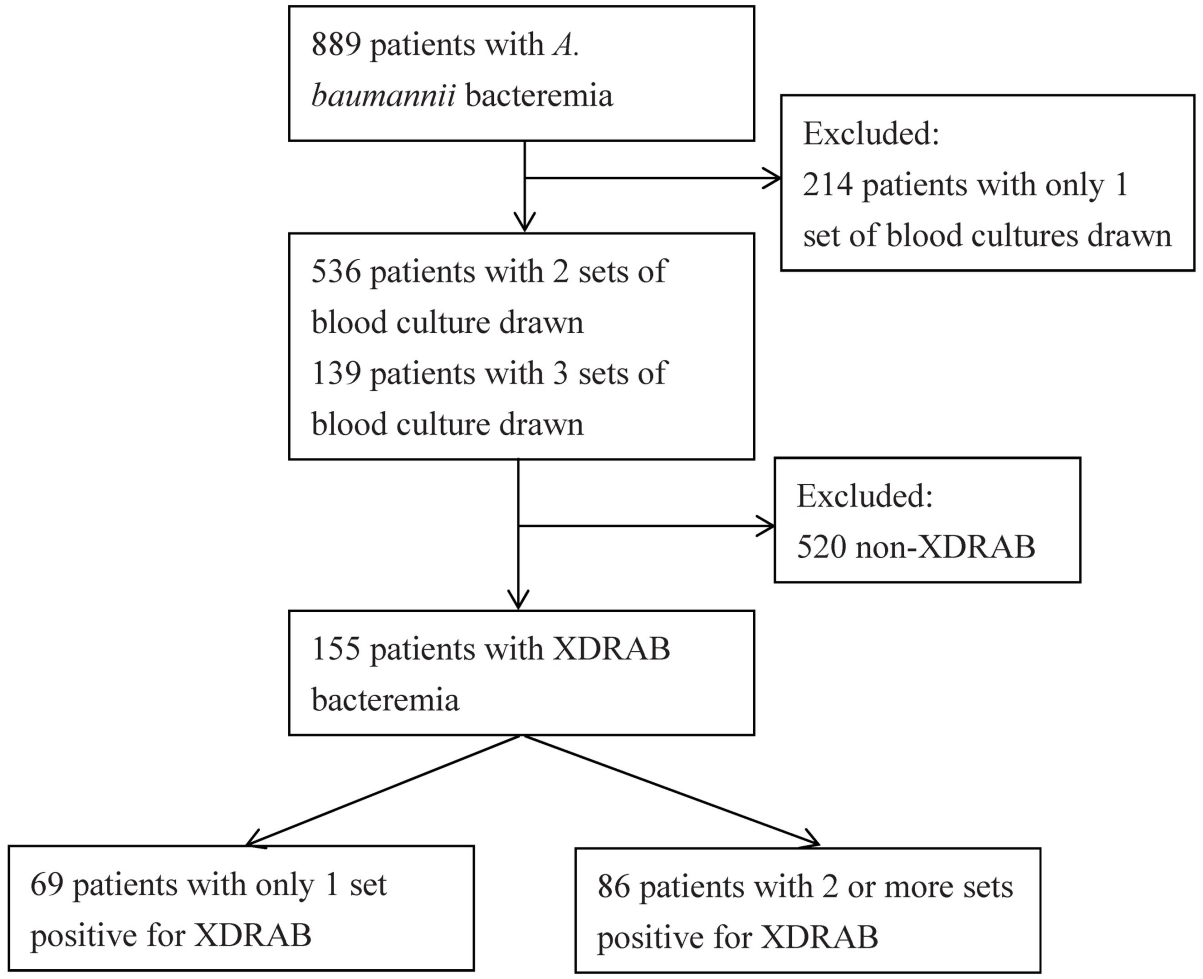
Flow diagram illustrating the selection of patients for the study.

A total of 155 patients were enrolled in the study period (July 2010–June 2015) (Fig 1). The mean (SD) age of the study cohort was 66.9 (17.4) years, the Pitt bacteremia score was 6.1 (3.3) points, the Charlson comorbidity index was 3.9 (3.0) points, and hospitalization duration before *A*. *baumannii* bacteremia onset was 31.7 (30.1) days. One hundred and four (67.1%) patients were male, 45 (29.0%) patients had underlying malignancy, and 30 (19.4%) patients received immunosuppressive therapy. The all-cause in-hospital mortality was 71.6%.

The patients’ characteristics differed considerably between those with single- versus multiple-positive blood cultures (Table 1). The single-positive blood culture group had a higher frequency of polymicrobial rather than monomicrobial infection (36.2% vs. 14.0%; *P* = 0.002). The multiple-positive blood culture group had a higher frequency of underlying malignancy (36.0% vs. 20.3%; *P* = 0.030), leukopenia (37.2% vs. 16.2%; *P* = 0.004), thrombocytopenia (56.0% vs. 26.5%; *P* < 0.001), and pneumonia (48.8% vs. 30.4%; *P* = 0.020), higher initial disease severity (as indicated by the Pitt bacteremia score, 6.6 vs. 5.5, *P* = 0.030), and correspondingly higher rates of 14-day (*P* = 0.006) and 28-day mortality (70.9% vs. 43.5%; *P* = 0.001).

**Table 1:** Demographics and clinical characteristics of patients with either single- or multiple-positive blood cultures positive for *A*. *baumannii*.

Variables^a^	Single-positive n = 69	Multiple-positive n = 86	*P* value
Age (years)	69.7 (15.7)	64.6 (18.5)	0.07
Age > 60 (years)	51 (73.9)	53 (61.6)	0.12
Male	42 (60.9)	62 (72.1)	0.17
Body mass index (Kg/m^2^)	24.3 (5.8)	24.1 (3.9)	0.82
Admission to onset (days)	33.3 (35.1)	30.4 (25.6)	0.55
Central venous catheter	57 (82.6)	69 (80.2)	0.84
Ventilator use	49 (71.0)	68 (79.1)	0.27
Polymicrobial bacteremia	25 (36.2)	12 (14.0)	0.002
Colistin use	42 (60.9)	40 (46.5)	0.11
**Previous antibiotics use**			
Prior carbapenem	30 (43.4)	50 (58.1)	0.08
Days of carbapenem	4.8 (7.1)	5.1 (6.7)	0.77
Prior anti-pseudomonal cephalosporin	50 (72.5)	61 (70.9)	0.86
Days of anti-pseudomonal cephalosporin	6.2 (6.1)	5.5 (5.8)	0.42
Prior anti-pseudomonal fluoroquinolones	23 (33.3)	37 (43.0)	0.25
Days of anti-pseudomonal fluoroquinolones	2.6 (4.8)	2.7 (3.9)	0.86
**Recruitment hospital**			
NTUH, Taipei Branch	45 (65.2)	47 (54.7)	0.20
NTUH, Yun-Lin Branch	8 (11.6)	19 (22.1)	
FEMH	16 (23.2)	20 (23.3)	
**Underlying diseases**			
Stroke	10 (14.5)	10 (11.6)	0.64
Myocardial infarction	5 (7.2)	3 (3.5)	0.47
Diabetes mellitus	2 (2.9)	6 (7.0)	0.30
Congestive heart failure	13 (18.8)	12 (14.0)	0.51
Chronic lung disease	6 (8.7)	12 (14.0)	0.45
Leukemia	4 (5.8)	7 (8.1)	0.76
Lymphoma	2 (2.9)	8 (9.3)	0.19
Metastatic cancer	3 (4.3)	4 (4.7)	0.99
Any malignancy	14 (20.3)	31 (36.0)	0.03
Steroid recipients	9 (12.0)	18 (20.9)	0.21
Immunosuppressive agent	13 (18.8)	17 (19.8)	0.99
Liver cirrhosis	8 (11.6)	18 (20.9)	0.14
End-stage renal disease	11 (15.9)	20 (23.3)	0.31
Charlson comorbidity index	3.4 (2.5)	4.3 (3.4)	0.09
**Disease severity at bacteremia onset**			
White blood cell (1000 cells/μL)	12.3 (8.8)	9.4 (9.6)	0.06
Leukopenia (<4000 cells/μL)	11 (16.2)	32 (37.2)	0.004
Hemoglobin (g/dL)	9.5 (1.6)	9.5 (1.8)	0.92
Platelet (10000 cells/μL)	17.8 (13.1)	12.0 (12.9)	0.007
Thrombocytopenia (<80000 cells/μL)	18 (26.5)	47 (56.0)	<0.001
Creatinine (mg/dL)	1.9 (1.3)	2.2 (1.8)	0.27
Total bilirubin (mg/dL)	4.5 (7.1)	5.4 (8.0)	0.49
Pitt bacteremia score	5.5 (3.1)	6.6 (3.5)	0.03
**Sites of infection**			
Pneumonia	21 (30.4)	42 (48.8)	0.02
Catheter-associated infection	17 (24.6)	16(18.6)	0.43
Urinary tract infection	3 (4.3)	13 (15.1)	0.03
Intra-abdominal infection	6 (8.7)	10 (11.6)	0.61
Surgical site infection	10 (14.5)	6 (7.0)	0.18
**Outcome**			
Overall in-hospital mortality	45 (65.2)	66 (76.7)	0.15
14-Day mortality	24 (34.8)	50 (58.1)	0.006
28-Day mortality	30 (43.5)	61 (70.9)	0.001
Early mortality before culture report	11 (15.9)	44 (51.2)	<0.001

^*a*^ Data are mean (standard deviation) for continuous variables and number of cases (percentage) for categorical variables, with two-tailed Student’s T test for the former and Fisher’s exact test for the later.

Among the 86 patients who had multiple-positive blood cultures, 12 had polymicrobial infections with the following co-pathogens (*n*): enterococci (8), staphylococci (1), *Escherichia coli* (1), *Klebsiella pneumoniae* (1), *Elizabethkingia meningosepticum* (1), *Stenotrophomonas maltophilia* (1), *Pseudomonas aeruginosa* (1). Among the 69 patients who had single-positive blood cultures, 25 had polymicrobial infections with the following co-pathogens (*n*): enterococci (8), staphylococci (6), *E*.*coli* (2), *K*.*pneumoniae* (2), *E*. *meningosepticum* (1), *S*. *maltophilia* (2), *Candida* spp. (2), *Bacteroides vulgatus* (1), *Acinetobacter lwoffii* (1), *Enterobacter cloacae* (1), *Burkholderia cepacia* complex (1), cryptococci (1), *Delftia acidovorans* (1). Six of the polymicrobial-infected patients (2 in the multiple-positive and 4 in the single-positive groups) had more than one co-pathogens isolated.

### Univariate analysis of factors associated with 28-day mortality

The multiple-positive group compared with the single-positive group had higher 28-day mortality by univariate logistic regression (odds ratio (OR) 3.17; *P* = 0.001) and by the Kaplan–Meier survival analysis (hazard ratio (HR) 2.29; log-rank *P* < 0.001). There were no major differences in the characteristics of patients who died within 28 days of bacteremia and those who did not, except for underlying chronic lung disease (OR 4.01; *P* = 0.03), prior carbapenem use (OR 2.38; *P* = 0.009), use of mechanical ventilation (OR 4.5; *P* < 0.001), the Pitt score (OR 1.43; *P* < 0.001), thrombocytopenia (OR 10.18; *P* < 0.001), and colistin-based treatment (OR 0.33; *P* = 0.001) by univariate analysis (Table 2). Non-survivors did not have a higher proportion of polymicrobial bacteremia compared to survivors (25.3% vs. 23.4%, *P* = 0.79) (Table 2). The mortality rates of patients co-infected with enterococci (12/16), staphylococci (4/7), or other pathogens (11/19) were not greater than that of monomicrobial XDRAB bacteremia (68/118) (*P* = 0.18, 0.98, and 0.98, respectively).

**Table 2:** Logistic-regression analysis for factors associated with 28-day mortality.

Risk factor^a^	Survivor (n = 64)	Mortality (n = 91)	Univariate		Multivariate^b^	
			Odds ratio [95% CI]	*P value*	Odds ratio [95% CI]	*P value*
Age (years)	66.7 (16.6)	67.1 (18.1)	1.00 [0.98–1.02]	0.88		
Age > 60 (years)	44 (68.8)	60 (65.9)	0.88 [0.44–1.74]	0.71		
Male	40 (62.5)	64 (70.3)	1.42 [0.72–2.80]	0.31		
Body mass index (Kg/m^2^)	24.0 (5.0)	24.3 (4.7)	1.01 [0.95–1.09]	0.71		
Multiple positive blood cultures	24 (37.5)	61 (67.0)	3.39 [1.74–6.61]	<0.001	2.34 [1.03–5.28]	0.04
Admission to onset (days)	33.1 (28.0)	30.7 (31.7)	1.00 [0.99–1.01]	0.62		
Central venous catheter	51 (79.7)	75 (82.4)	1.19 [0.53–2.70]	0.67		
Ventilator use	38 (59.4)	79 (86.8)	4.50 [2.05–9.88]	<0.001		
Polymicrobial bacteremia	15 (23.4)	23 (25.3)	1.10 [0.52–2.33]	0.79		
Prior carbapenem use	25 (39.1)	55 (60.4)	2.38 [1.24–4.59]	0.009		
Days of carbapenem use	4.5 (7.0)	5.3 (6.8)	1.02 [0.97–1.07]	0.49		
Prior anti-Pa cephalosporin use	41 (64.1)	70 (76.9)	1.87 [0.92–3.79]	0.08		
Days of anti-Pa cephalosporin use	6.0 (6.6)	5.7 (5.4)	0.99 [0.94–1.05]	0.72		
Prior anti-Pa FQs use	24 (37.5)	36 (39.6)	1.09 [0.57–2.11]	0.80		
Days of anti-Pa FQs use	2.9 (4.9)	2.6 (3.8)	0.98 [0.91–1.06]	0.65		
Recruitment hospital						
NTUH, Taipei Branch	46 (71.9)	46 (50.6)	reference			
NTUH, Yun-Lin Branch	7 (10.9)	20 (22.0)	2.86 [1.10–7.41]	0.03		
FEMH	11 (17.2)	25 (27.5)	2.27 [1.00–5.15]	0.05		
Underlying diseases						
Stroke	8 (12.5)	12 (13.2)	1.06 [0.41–2.77]	0.90		
Myocardial infarction	6 (9.4)	2 (2.2)	0.22 [0.04–1.11]	0.07		
Diabetes mellitus	4 (6.3)	4 (4.4)	0.69 [0.17–2.87]	0.61		
Congestive heart failure	11 (17.2)	14 (15.4)	0.88 [0.37–2.08]	0.76		
Chronic lung disease	3 (4.7)	15 (16.5)	4.01 [1.11–14.50]	0.03		
Leukemia	2 (3.1)	9 (9.9)	3.40 [0.71–16.31]	0.13		
Lymphoma	2 (3.1)	8 (8.8)	2.99 [0.61–14.56]	0.18		
Metastatic cancer	2 (3.1)	5 (5.5)	1.80 [0.34–9.59]	0.49		
Any malignancy	14 (21.9)	31 (34.1)	1.85 [0.89–3.85]	0.10		
Steroid recipients	7 (10.9)	20 (22.0)	2.29 [0.91–5.81]	0.08		
Chemotherapy	5 (7.8)	13 (14.3)	1.97 [0.66–5.82]	0.22		
Immunosuppressive agent	9 (14.1)	21 (23.1)	1.83 [0.78–4.32]	0.17		
Liver cirrhosis	6 (9.4)	20 (22.0)	2.72 [1.03–7.22]	0.04		
End-stage renal disease	11 (17.2)	20 (22.0)	1.36 [0.60–3.07]	0.46		
Charlson comorbidity index	3.5 (2.5)	4.2 (3.3)	1.07 [0.96–1.21]	0.20		
Disease severity at bacteremia onset						
White blood cell (1000 cells/μL)	12.0 (7.7)	9.7 (10.3)	0.97 [0.94–1.01]	0.14		
Leukopenia (<4000 cells/μL)	9 (14.1)	34 (37.8)	3.71 [1.63–8.45]	0.002		
Hemoglobin (g/dL)	9.9 (1.6)	9.4 (1.9)	0.86 [0.71–1.03]	0.11		
Platelet (10000 cells/μL)	21.2 (13.3)	9.9 (11.2)	0.92 [0.89–0.95]	<0.001	0.94 [0.91–0.98]	0.001
Thrombocytopenia (<80000 cells/μL)	9 (14.1)	56 (62.9)	10.18 [4.46–23.27]	<0.001		
Creatinine (mg/dL)	1.9 (1.5)	2.2 (1.9)	1.11 [0.91–1.35]	0.29		
Total bilirubin (mg/dL)	2.8 (5.2)	6.2 (8.4)	1.10 [1.01–1.19]	0.03		
Pitt score	4.2 (2.9)	7.5 (3.0)	1.43 [1.26–1.63]	<0.001	1.33 [1.16–1.53]	<0.001
Sites of infection						
Pneumonia	22 (34.4)	41 (45.1)	1.57 [0.81–3.03]	0.18		
Catheter-associated BSI	16 (25)	17 (18.7)	0.69 [0.32–1.49]	0.35		
Urinary tract infection	6 (9.4)	10 (11.0)	1.19 [0.41–3.47]	0.75		
Intra-abdominal infection	3 (4.7)	13 (14.3)	3.39 [0.92–12.43]	0.07		
Surgical site infection	3 (4.7)	13 (14.3)	3.39 [0.92–12.43]	0.07		
Colistin use	44 (68.8)	38 (41.8)	0.33 [0.17–0.64]	0.001	0.44 [0.19–0.99]	0.05

^*a*^ Data are mean (standard deviation) for continuous variables and number of cases (percentage) for categorical variables

^*b*^ Multivariate logistic regression model: *n* = 155, Nagelkerke/Cragg & Uhler's R-squared = 0.454, Pearson goodness-of-fit test *P* = 0.15 > 0.05, estimated area under the ROC curve = 0.8568

### Multivariate analysis of factors associated with 28-day and early mortality

**Fig 2:**
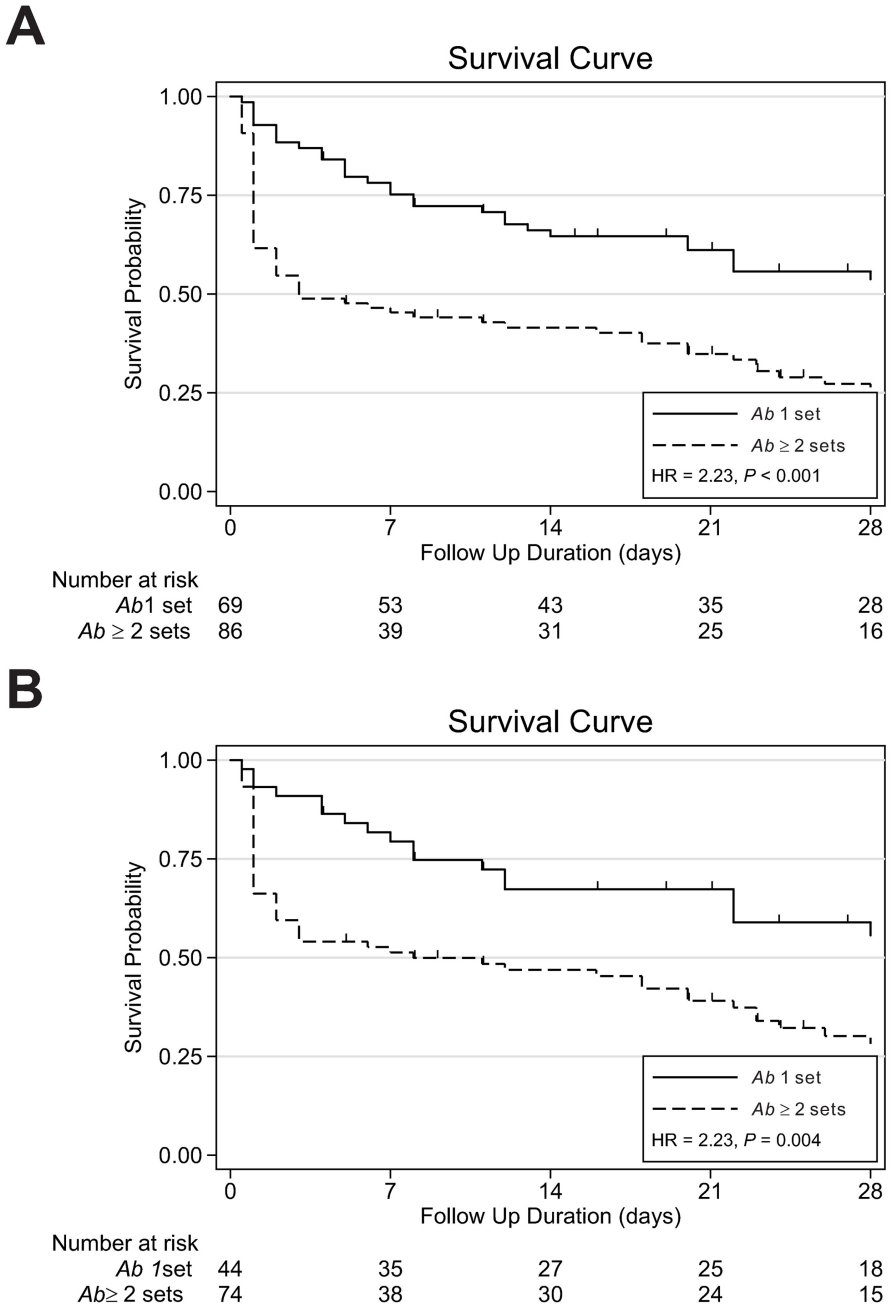
Kaplan-Meier stratified by 1 set and ≥2 sets blood cultures positive for *A*. *baumannii* showing inferior survival of ≥2 sets blood cultures positive for *A*. *baumannii* compared to 1 set of *A*. *baumannii* among all *A*. *baumannii* bacteremic patients (A) and among mono-microbial *A*. *baumannii* bacteremic patients (B).

We adjusted for the above covariates and also those variables in the univariate analysis with a *P* < 0.2 in the multivariate logistic regression analysis (Table 2). Excess 28-day in-hospital mortality was predicted by multiple-positive blood cultures (adjusted OR (aOR), 2.34; 95% confidence interval (CI), 1.03–5.28; *P* = 0.04) (Table 2) by logistic regression. By multivariate Cox proportional-hazard analysis, multiple-positive blood cultures (adjusted HR (aHR), 1.80; 95% CI, 1.13–2.85; *P* = 0.010), Pitt bacteremia score (aHR, 1.17; 95% CI, 1.09–1.26; *P* < 0.001) were independent risk factors for mortality, whereas each 10,000/mL increment in platelet count (aHR, 0.95; 95% CI, 0.92–0.97; *P* < 0.001) was associated with survival. The Kaplan–Meier curves in Fig 2A showed that the multiple-positive blood cultures group experienced higher mortality that was greatest in the early period (within 3 days) compared to the single-positive blood culture group.

We further analyzed the risk for early mortality. Among the 155 patients, 55 patients died rapidly after the onset of bacteremia, typically before the results of blood cultures became known. Early mortality was independently predicted by higher Pitt scores (aOR, 1.38; 95% CI, 1.17–1.63; *P* < 0.001), leukopenia (aOR 4.84; 95% CI, 1.58–14.86: *P* = 0.006), thrombocytopenia (aOR, 5.04; 95% CI, 1.82–13.98; *P* = 0.002) and multiple-positive blood cultures (aOR, 4.52; 95% CI, 1.67–12.26; *P* = 0.003).

### Sensitivity analysis of monomicrobial-infected patients

Excluding those with polymicrobial infections, there were 118 monomicrobial-infected patients included in the sensitivity analysis. Eighteen of the 44 (40.9%) patients with single-positive culture died within 28-days compared to 50 of 74 (67.6%) with multiple-positive cultures (*P* = 0.007). Among monomicrobial-infected patients, multiple sets of positive blood cultures were consistently associated with mortality (aOR, 4.37; 95% CI, 1.22–15.72; *P* = 0.02), independently of the Pitt score (aOR, 1.55; 95% CI, 1.24–1.95; *P* < 0.001) and colistin use (aOR, 0.18; 95% CI, 0.05–0.72; *P* = 0.02). The Kaplan-Meier survival curves of the multiple-positive and single-positive groups of the monomicrobial-infected subgroup in Fig 2B similarly show a more rapid and deep drop for the patients with multiple-positive cultures.

## Discussion

To our knowledge, this is the first study to compare the clinical outcomes of patients with single- versus multiple-positive blood cultures of XDRAB. Patients who had multiple sets of positive blood cultures for XDRAB differed significantly from those who had only one set of positive cultures out of multiple samples. Although single-positive bacteremia patients did not have negligible mortality, multiplicity of positive blood cultures independently predicted greater and more rapid mortality. These findings imply that the blood culture positivity rate can be used as a simple prognostic indicator in the context of XDRAB bacteremia and further studies to examine more efficient methods to detect contamination versus true infection in the context of a single-positive blood culture are needed.

The differences in mortality between single versus multiple-positive patient groups might be explained by higher bacterial load since semi-quantitative blood cultures have been shown to be a measure of the magnitude of *S*. *aureus* bacteremia [18] and Chuang *et al*. have shown that the bacterial burden measured by real-time PCR is associated with the in-hospital mortality of *A*. *baumannii* bacteremia [17]. Compared to the expensive molecular quantification of the latter study, the semi-quantitative blood culture has the advantages of routine accessibility without additional costs or labor and remains the gold standard for the diagnosis of bacteremia [28, 29]. However, single-positive blood cultures do not necessarily predict better outcomes compared to multiple-positive blood cultures in the case of coagulase-negative staphylococci (CoNS) and enterococci (Table 3). In addition, the difference between the single versus multiple-positive patient groups might also be explained by the lack of distinction between contamination and true infection.

**Table 3:** Review of studies post-1990s comparing the associated mortality of single-positive (SP) versus multiple-positive (MP) blood cultures based on the identity of the microorganism.

Ref.	Date	Total (n)	SP (n)	MP (n)	Microorganism	Crude mortality of SP vs. MP (%)	Multivariate analysis of mortality risk as predicted by culture positivity	Comments
[20]	2005	234	163	71	Coagulase-negative staphylococci	27.0 *vs*. 18.3	RR 1.9 for SP (95% CI 0.9–3.8), *p* = 0.079	The SP group had higher crude mortality rates than the MP group. Baseline differences between SP and MP in the univariate analysis included ICU stay (59.5% *vs*. 38%, p = 0.002, shorter duration between admission and bacteremia (11 *vs*. 15 days, p = 0.025, blood drawn through catheter (63.8% *vs*. 45.1%, p = 0.008), longer time to positive (3 *vs*. 2 days, p = 0.001), considered contaminant by physician in charge (68.7% vs. 4.2%, p = <0.001), adequate antibiotic therapy (32.5% v 81.7% p = <0.001).
[35]	2011	54	32	22	Coagulase-negative staphylococci	18.8 *vs*. 18.2	Not performed.	There was no significant correlation between number of bottles positive for culture and mortality.
[19]	2014	189	95	94	Enterococci (VRE 39.7%)	17.9 *vs*. 19.1	OR 1.00 (95% CI 0.42–2.40), *p* = 0.993	There was no significant correlation between number of bottles positive for culture and in-hospital mortality or elimination.
[36]	2016	446	214	232	Enterococci	12.5 *vs*. 17.2	HR for MP 1.89 (95% CI 1.13–3.16), *p* < .10	The SP group had lower attributable mortality than the MP group but similar crude mortality. Differences between SP and MP in the univariate analysis: skin as primary source of infection (20.6% *vs*. 7.3%, p = 0.003, infective endocarditis (3.3% *vs*. 12.6%), p = 0.004), nosocomial BSI (51.9% *vs*. 38.4%, p = = 0.012), VRE (56.1% *vs*. 40.9%, p = 0.001), *E*. *fecium* (41.1% *vs*. 23.8%, p<0.001), decreased median inpatient survival for MP (65 *vs*. 82 days).
This study	2017	155	69	86	*Acinetobacter baumannii* (XDRAB)	65.2 *vs*. 76.8	OR of 28-day mortality for MP = 2.34 (95% I 1.03–5.28), p = 0.04	The MP group had higher rates of 14-day (*p* = .006) and 28-day mortality (70.9% *vs*. 43.5%; *p* = .001). The MP group in univariate analysis had a lower frequency of polymicrobial infection (14.0% *vs*. 36.2%; *p* = .002) and higher frequency of underlying malignancy (36.0% *vs*. 20.3%; *p* = .03), leukopenia (37.2% *vs*. 16.2%; *p* = .004), thrombocytopenia (56.0% *vs*. 26.5%; *p* < .001), and pneumonia (48.8% *vs*. 30.4%; *p* = .02), higher Pitt score, 6.6 *vs*. 5.5, *p* = .03).

Despite its limitations, the blood culture remains the “gold standard” for the detection of bacteremia and at least 2 blood cultures should be sampled simultaneously before therapy according to the US Centers for Disease Control and Prevention/National Health Surveillance Network (CDC/NHSN) and Surviving Sepsis Campaign [22, 30]. Suggested laboratory criteria for true bacteremia include growth within 48h and multiple cultures positive for the same organism [31–33]. In contrast, increased duration before positivity, polymicrobial growth of skin organisms, or growth during treatment suggest contamination [31, 32]. In this study, we confirmed that polymicrobial infections were more common among single-positive rather than multiple-positive groups and that the former had less severe disease and mortality compared to the latter. However, the crude mortality rate of the single-positive group in this study was 65% which is much higher than the reported pooled crude mortality rate of 27% for nosocomial bacteremia from a concurrent surveillance study of 49 US hospitals or the reported rates of 19–27% and 12.5–17.5% for bacteremia with single-positive blood cultures for CoNS [20, 34, 35] and enterococci, respectively (Table 3) [19, 36]. In addition, the mean Pitt score in our single-positive group was high (5.5) making it unlikely that the majority of single-positive patients had pseudobacteremia and thus, better outcomes.

Unlike CoNS and enterococci, *A*. *baumannii* is not considered a skin contaminant [22]. Thus, growth of XDRAB from a single blood culture will tend to prompt treatment. However, given secular changes in the standards of care, it is plausible that *A*. *baumannii* could occasionally contaminate cultures drawn from central lines in contemporary ICUs. In Weinstein’s landmark studies of 500 episodes, later expanded to 843 episodes of positive blood cultures in hospital inpatients published in 1983 and 1997, respectively [21, 37], upon which current understanding of blood culture results that are incorporated in widely used CDC/NHSN definitions for laboratory-confirmed bloodstream infections are based, species thought to represent true infections when isolated from a blood culture included *S*. *aureus*, pneumococcus, *E*. *coli* and other *Enterobacteriaceae*, *P*. *aeruginosa*, and *C*. *albicans*, whilst species thought to represent contamination in a significant proportion of cases, included CoNS, *Corynebacterium* species, *Bacillus* species, *Propionibacterium acnes*, *Micrococcus species*, viridans group streptococci, enterococci, and *C*. *perfringens*. Yet at the time, *Acinetobacter* species were rarely isolated, being only identified in 0–1% (16 of 1844 blood isolates) in Weinstein’s studies, in 0–1% (4 of 348 blood isolates) among male veterans, and 1.3% (67 of 5058 blood isolates) among North American 1997 SENTRY participants [38–40]. Since then, the isolation rate of *Acinetobacter* species from blood cultures has increased significantly, particularly in the ICU setting, however it is not known what proportion represents true bacteremia or contamination.

Furthermore, there have been few studies in the last two decades to document the clinical significance of single versus multiple blood cultures for organisms other than CoNS and enterococci (Table 3). To our knowledge, our study is the first to document the prognostic impact of the blood culture positivity rate for gram-negative species. Further studies on whether the culture positivity rate impacts the outcomes of microorganisms not traditionally considered as skin contaminants, to reflect the changing microbiology and epidemiology are warranted.

Although treated equivalently as true bacteremia, we found major differences in the clinical characteristics between patient groups, namely single-positive patients were less likely to have monomicrobial bacteremia, underlying malignancy, pneumonia, bicytopenia, high Pitt scores and fatal disease compared to multiple-positive patients. These findings question the uniformity of treating single-positive patients as multiple-positive patients and of enrolling such patients together in clinical trials.

Currently registered trials for multidrug resistant *A*. *baumannii* at ClinicalTrials.gov all employ culture-confirmed randomization without specifying the number of positive blood cultures. Such trials will miss a significant proportion (51.2%) of multiple-positive patients and preferentially enroll those with minor disease or pseudobacteremia (84.1%). Consequently, such trials cannot be relied on to give response and mortality rates that can be replicated in the real world. For example, colistin use was independently associated with survival in this study. While this has been attributed to its effectiveness, it may reflect the fact that those who had already survived to when cultures were reported were patients with mild disease. After excluding the early mortality cases, colistin use was no longer associated with survival (27/71 (38.0%) vs. 9/29 (31%), *P* = 0.65). Our findings therefore support the importance of empiric randomization for maximizing the likelihood of detection of true superiority of the investigational agent for XDRAB [41].

Given that multiple-positivity was associated with increased mortality that was greatest in the period before culture becomes known and interventions can be initiated, we analyzed whether there were associated factors that could be manipulated to decrease the rates of multiple-positive blood cultures. Considering the modifiable risk factors such as length of hospital stay, central venous catheter and ventilator use, immunosuppression and the cumulative use of antimicrobial agents within 28 days of bacteremia onset, we found that carbapenem exposure independently predicted multiplicity of positive blood cultures for XDRAB (aOR, 2.80; 95% CI, 1.03–7.64; *P* = 0.045). Therefore, judicious carbapenem use might decrease the rate of multiple-positive blood cultures rates and curb the associated early mortality.

The strengths of the current study include the prospective multicenter design and the use of a hard end-point, mortality, to avoid recall biases. We included only patients who had more than two simultaneous blood cultures to avoid the heterogeneity of previous studies that mixed single-positive out of a total of one set of blood culture sample with single out of multiple sets of blood culture and also avoided the mixing of multiple-positive cultures due to sequential positive cultures (representing persistent bacteremia). We also validated our findings by sensitivity analyses. However, the following limitations should be acknowledged. First, the cohort studied may not be sufficiently large to render non-observed confounders obsolete. Since randomization may be impractical, our findings should be corroborated by a larger study. Second, we excluded a significant portion (214/889) of patients who had only one set of blood cultures collected. Third, we did not compare the bacterial loads of XDRAB in the single and multiple-positive patients by quantitative blood culture or real-time PCR. We also did not measure the bacterial loads of other co-pathogens. Fourth, there was no control group for the patients with a single-positive blood culture. Hence, what proportion of single-positive patients had pseudobacteremia and whether the 28-day mortality rate of 43.5% for this group is in excess of the mortality of critically-ill patients without XDRAB bacteremia remain unanswered.

## Conclusions

In conclusion, the number of positive blood cultures is a prognostic indicator of the severity of XDRAB bacteremia. Multiple- compared to single-positive blood cultures for XDRAB is associated with rapidly fatal disease. In order to curb the alarming early mortality rates, prevention of multiple-positive XDRAB bacteremia by carbapenem stewardship might be a better strategy than attempts at curing the infection after culture results become known. Lastly, more research is needed to discern true infection and contamination among patients with single-positive cultures.

## Supporting information

S1 Spreadsheet Containing the data used in this study. (XLSX)
